# *POLE/POLD1* mutation and tumor immunotherapy

**DOI:** 10.1186/s13046-022-02422-1

**Published:** 2022-07-02

**Authors:** Xiaoting Ma, Lin Dong, Xiu Liu, Kai Ou, Lin Yang

**Affiliations:** 1 Department of Medical Oncology, National Cancer Center/National Clinical Research Center for Cancer/Cancer Hospital, Chinese Academy of Medical Sciences and Peking Union Medical College, Beijing, 100021 China; 2 Department of Pathology, National Cancer Center/National Clinical Research Center for Cancer/Cancer Hospital, Chinese Academy of Medical Sciences and Peking Union Medical College, Beijing, 100021 China

**Keywords:** Immune checkpoint inhibitor, MSI, POLD1, POLE, Tumor, Tumor mutation burden

## Abstract

*POLE* and *POLD1* encode the catalytic and proofreading subunits of DNA polymerase ε and polymerase δ, and play important roles in DNA replication and proofreading. *POLE/POLD1* exonuclease domain mutations lead to loss of proofreading function, which causes the accumulation of mutant genes in cells. *POLE/POLD1* mutations are not only closely related to tumor formation, but are also a potential molecular marker for predicting the efficacy of immunotherapy in pan-carcinomatous species. The association of *POLE/POLD1* mutation, ultra-high mutation load, and good prognosis have recently become the focus of clinical research. This article reviews the function of *POLE/POLD1*, its relationship with deficient mismatch repair/high microsatellite instability, and the role of *POLE/POLD1* mutation in the occurrence and development of various tumors.

## Background

The incidence rate and mortality rate of cancer is increasing worldwide. In 2020, 19.3 million new cancer cases and 10 million cancer deaths occurred worldwide [1]. Cancer is one of the most common causes of death worldwide, and cancer prevention and treatment have thus become a major public health concern. Traditional cancer treatment methods, including surgery, radiotherapy, and chemotherapy, have limitations. An improved understanding of the tumor microenvironment and tumor immune mechanisms in recent years led to the development of tumor immunotherapy. Immunotherapy, represented by immune checkpoint inhibitors (ICIs), has improved the long-term survival of patients with advanced solid tumors such as lung cancer, melanoma, colorectal cancer, and esophageal cancer. Among them, programmed death receptor 1 (PD-1) antibody is the most widely used. Studies have confirmed that compared with traditional chemotherapy and targeted therapy, sustained remission is possible even after discontinuing ICI treatment. The 5-year overall survival rate of patients with non-small cell lung cancer (NSCLC) treated with ICIs increased to 16% [2]. However, the efficacy of ICIs is not satisfactory. A clinical study involving multiple cancer immunotherapies showed that only 20 − 40% of patients responded to ICI [3]. Therefore, it is important to identify additional reliable molecular markers for predicting the outcome of ICI treatment, which would allow the identification of patients who would benefit from ICI treatment, thereby decreasing overtreatment.

Deficient mismatch repair (dMMR)/high microsatellite instability (MSI-H) was the first identified molecular marker for predicting the efficacy of ICIs [4]. In 2017, Le et al. conducted a clinical study of dMMR patients with 12 tumor types, which showed that the objective response rate (ORR) of anti-PD-1 treatment for dMMR advanced cancer was 53%, and 21% of patients achieved complete remission [5]. Although new detection techniques have been proposed to improve the sensitivity of microsatellite fragment detection, the proportion of dMMR/MSI-H is still low in current clinical practice [6]. High tumor mutation burden (TMB-H) is the second molecular marker for predicting the efficacy of ICIs in pan-cancer species. Several studies have shown that it is significantly related to the benefit of immunotherapy [7, 8]. In 2019, MSKCC released the largest study of TMB predicting the effect of immunotherapy so far, analyzed the data of 7,033 cancer patients of 10 cancer species, and confirmed that TMB-H was related to better overall survival (OS) after receiving immunotherapy. In addition, there are many biomarkers related to the efficacy of ICIs, such as PD-L1 expression, epigenetic changes, somatic copy number changes, and intestinal microflora [9–11]. However, these biomarkers have their own limitations; therefore, it is important to explore effective markers for predicting the efficacy of ICIs. *POLE/POLD1* mutation is not only closely related to tumor formation, but also a potential molecular marker for predicting the efficacy of immunotherapy in pan-cancer species. In this article, the function of *POLE/POLD1* and the effect of *POLE/POLD1* mutation on tumorigenesis, prognosis, and the effect of immunotherapy are reviewed.

### Functions of *POLE/POLD1*

DNA polymerase ε (Pol ε) and polymerase δ (Pol δ) both belong to the DNA polymerase B family, which have polymerase activity and 3′–5′ exonuclease activity. Pol ε and Pol δ can accurately select a base complementary to the template chain to extend the DNA chain and guide the synthesis of the DNA leading and lagging strands [12, 13]. In addition, Pol ε and Pol δ recognize and repair the mismatched bases through the proofreading activity of its exonuclease region [14]. Pol ε and Pol δ also function in nucleotide excision repair and double strand break repair [15, 16]. The exonuclease domains of *POLE* and *POLD1* have the highest homology, with 23% homology and 37% similarity [17]. Therefore, most studies analyze the two genes at the same time. *POLE*, also known as *POLEl*, *FILS*, and *CRCSl2*, is located in human chromosome 12q24.3. Its encoding product POLE, the largest subunit of Pol ε, contains 2286 amino acids and has a molecular weight of 262 kDa. *POLE* encodes the catalytic and collation subunits of Pol ε. *POLD1* is located in 19q13.3-q13.4 and is the encoding gene of the Pol δ catalytic subunit. Its molecular weight is 126 kDa, and it can encode 1,107 amino acid residues. Pol δ expression was consistent with its involvement in DNA replication, suggesting that Pol δ expression was related to the state of proliferation. The exonuclease domain of *POLE/POLD1* recognizes and removes wrong bases generated during replication. Therefore, exonuclease domain mutation of *POLE*/*POLD1* (*POLE*/*POLD1*-EDM) lead to the loss of proofreading function, resulting in the accumulation of mutated genes in cells. In addition, there are three auxiliary subunits in eukaryotes, POLE2, POLE3, and POLE4, which play important roles in cell cycle regulation [14].

In eukaryotes, an error occurs for every 10^9^ to 10^10^ nucleotides of DNA replication. *POLE/POLD1* not only participates in DNA replication, but also plays a critical role in maintaining the fidelity of DNA replication [18]. *POLE/POLD1-*EDM causes a 10–100-fold increase in the mutation rate during DNA replication [19]. Albertson et al. introduced a mutation into the exonuclease domain of POLE by gene homologous recombination and compared the somatic mutation rates of homozygous mutant mice, heterozygous mutant mice, and wild-type mice in the exonuclease domain of *POLE*. The results showed that the *POLE* homozygous mutation increased the somatic mutation rate of the mouse genome and the incidence of sporadic tumors in mice. The main symptoms were small intestinal adenoma and adenocarcinoma, and the median survival was lower in *POLE*-mutant mice than in *POLE* wild-type mice. There was no difference in the survival rate between heterozygous mutant mice and wild-type mice, indicating that the *POLE* mutation had the greatest impact on the proofreading function after homozygous deletion [20]. *POLE* somatic or germline mutations are found in many tumors, including non-melanoma skin cancer, endometrial cancer (EC), colorectal cancer (CRC), melanoma, bladder cancer, esophageal cancer, and lung cancer [21]. Wang et al. analyzed 47,721 patients with different cancer types and showed that the incidence of *POLE* and *POLD1* somatic mutations was 2.79% and 1.37%. *POLE/POLD1* germline mutation is a predisposing factor for CRC, EC, ovarian cancer, and brain tumors [22].

### Correlation between *POLE/POLD1* mutation and TMB and MSI

**Fig. 1:**
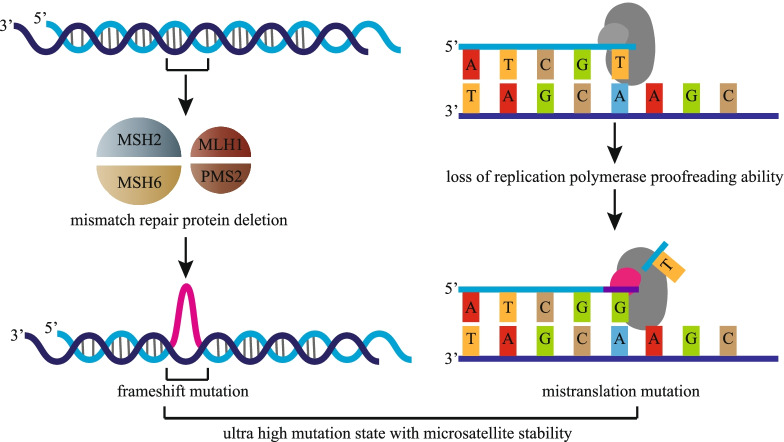
Ultra high mutation state of microsatellite stability. The most common mutation in MSI-H tumors is frameshift deletion mutation, whereas the most common mutation in *POLE/POLD1*-mutant tumors is missense mutation. The loss of mismatch repair ability combined with the loss of replication polymerase proofreading ability can produce defects of full replication repair, resulting in ultra-high mutation with MSS

**Fig. 2:**
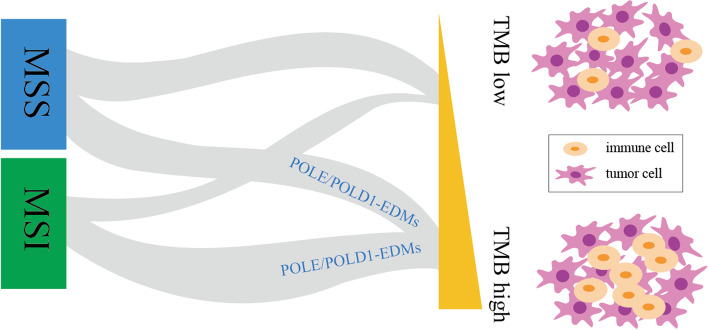
Correlation between* POLE/POLD1 *mutation and TMB and MSI. The TMB of *POLE/POLD1* mutations referred to as ultra-high mutation, which is associated with MSS. However, not all *POLE/POLD1* mutations are MSS. Tumors with high TMB usually have stronger T lymphocyte infiltration and can exert stronger anti-tumor activity

DNA mismatch repair and DNA polymerase proofreading are two main mechanisms to ensure the fidelity of genome replication. These two mechanisms can occur independently or simultaneously. The most common mutation in MSI-H tumors is frameshift deletion mutation, whereas the most common mutation in *POLE*-mutant tumors is missense mutation [23]. Tumors with dMMR/MSI-H or *POLE* mutations usually show high TMB [23]. MSI-H is mainly limited to tumors in the range of 10–100 mut/Mb, whereas the TMB of *POLE/POLD1* mutations can exceed 100 mut/Mb, also known as ultra-high mutation, which is associated with microsatellite stability (MSS). The loss of mismatch repair ability combined with the loss of replication polymerase proofreading ability can produce defects of full replication repair, resulting in ultra-high mutation with MSS (Fig. 1) [24]. However, not all *POLE/POLD1* mutations are MSS. The Cancer Genome Atlas (TCGA) considers *POLE* mutation and MSI as two important indicators of molecular typing, and many studies have confirmed the above views. In a study of EC, *POLE* mutation and MSI, as two independent indicators, were classified into ultra-high mutation phenotype and strong mutation phenotype, respectively (Fig. 2). In CRC, Carethers et al. classified *POLE*-mutant tumors and MSI tumors as strongly mutated phenotypes [25]. Church et al. found that MSI and *POLE* mutation did not occur in the same tumor at the same time. The incidence of MSI in EC is 18.5%, whereas all ECs with *POLE* mutations present with MSS [26]. Meng et al. performed immunohistochemical staining of four mismatch repair proteins (MLH1, PMS2, MSH2, and MSH6) and showed that only one of the eight cases of *POLE*-mutant EC had the MSH6 deficiency, whereas 46.6% of wild-type *POLE* EC had dMMR [27]. Some studies have shown opposite results. A study of 544 cases of EC with *POLE* mutation showed that the proportion of MSS and MSI was similar, and MSI lacking MLH1 methylation was the most common type. This suggests that *POLE* could be used as a candidate mutant gene for screening for Lynch Syndrome [28]. In 2020, Mo et al. found that CRC with *POLE*-EDMs was more prone to MSI-H, and all patients had high TMB, with an average of 200.8 mut/Mb [29]. In 2019, proteomic studies showed that colon cancer with MSI-H was mainly enriched in mismatch repair pathways and *POLE* and *BRAF* mutations [30].

For tumors with both *POLE* mutation and MSI-H, the sequence of *POLE* mutation and dMMR is a research hotspot. Most MSI cases are caused by methylation of the MLH1 promoter. If dMMR precedes *POLE* mutation, similar proportions of MLH1 methylation can be expected in MSI patients of the *POLE*-wild-type and *POLE*-mutant type. However, wild-type *POLE* patients account for more than 80% of MLH1-silenced patients, and mutant *POLE* patients account for less than 50% of MLH1-silenced patients, providing opposing evidence for the MSI priority model [31]. Temko et al. explored the timing of *POLE* mutations during cell carcinogenesis and found that *POLE* mutations could also be detected in precursors of *POLE*-mutated tumors [32]. In addition, mutation of the tumor suppressor gene *PTEN* is considered to be an early event in the pathogenesis of EC. In the analysis of pathogenic *PTEN* mutations in EC, researchers found that *PTEN* mutations were more prevalent in *POLE*-mutated tumors than in *POLE*-wild-type tumors with pMMR or dMMR [33]. In addition, because dMMR may occur later than other mutation processes, it can be wrongly identified as MSS at an early stage. Therefore, the pathogenic somatic *POLE* mutation is considered an early or even initial mutation, and its accompanying mutant phenotype determines the unique carcinogenic pathway. Repeat assessment of microsatellite status at a later stage of the disease may yield different results from those obtained initially, which may provide a new direction for clinical diagnosis and treatment. Hwang et al. observed that the deficiency of MSH6 and MSH2 precedes *POLE* mutations. These authors suggested that *POLE* mutation was a secondary mutation to dMMR, leading to a significant increase of TMB in tumors with concurrent dMMR and *POLE* mutations through their effects on POLE function [23]. To further clarify the interaction between *POLE* mutations and MSI and their effect on tumorigenesis, He et al. divided tumors into different categories according to the clone ratio of *POLE* mutations. They found that *POLE*-mutated tumors were more prone to MSI-H than wild-type *POLE* tumors [34]. In conclusion, current evidence supports that *POLE* mutations are a driver of dMMR in tumors with both *POLE* mutations and MSI-H, ultimately leading to the occurrence of MSI-H.

### Pathogenic and non-pathogenic mutations of *POLE/POLD1*

Most current studies indicate that *POLE* mutation is related to ultra-high mutation load. However, some studies do not support this theory. Hwang et al. analyzed the *POLE*-mutant phenotype and found that tumors with a large number of *POLE* mutations do not show an abnormally high TMB [23]. These authors further explored the associated immune microenvironment and found that many *POLE* mutations may be non-functional mutations that fail to initiate an immune response [23].

The location of *POLE* mutations may be the dominant factor determining tumor phenotype and clinical outcome. *POLE*-EDMs can cause loss of proofreading function, resulting in the accumulation of mutated genes in cells. However, not all mutation sites in the exonuclease domain are pathogenic. The five most common mutation sites are P286R, V411L, S297F, A456P, and S459F, and are known as hotspot mutations [35]. To clarify the mutation site that best defines the pathogenic mutation of *POLE*, Castillo et al. analyzed 530 EC cases from TCGA including 82 cases with *POLE* mutations, of which 59 were in the exonuclease domain and 23 were outside the exonuclease domain. The results showed that EC cases with one of the five hotspot mutations had a characteristic genome sequence that differed from that of wild-type *POLE* EC with MSI-H or MSS. The researchers developed a practical scoring system by measuring the proportion of TMB, C > A, T > G, C > G, and used index scales as the scoring criteria (C > A over 20% = 1, T > G over 4% = 1, indels below 5% = 1, C > G below 0.6% = 1, TMB over 100 mut/Mb = 1, recurrent variant in EC = 1). To define the threshold of pathogenicity, the *POLE* scoring system was applied to cases with *POLE* hotspot mutation, *POLE* non-hot spot mutation, and *POLE*-wild-type in TCGA. Of the 41 cases with *POLE* hotspot mutation, 38 had a score > 5, and the remaining 3 cases had V411L mutation with a score of 4, whereas the *POLE*-EDM scores of non-hot spot mutations were lower. Therefore, a score ≥ 4 was used to define the pathogenicity of *POLE* mutations, a score ≤ 2 was classified as non-pathogenic mutations, and a score of 2–4 was defined as uncertain mutations [35].

In addition, a non-exonuclease domain mutation of *POLE/POLD1* has been found in 3 − 4% of CRCs and ECs [17]. Although there is not enough evidence to support its pathogenic role, a small number of studies have confirmed the pathogenic mutation of *POLE* outside the exonuclease domain. Some studies have confirmed that the V1368M mutation of *POLE* destroyed the function or structure of the protein and was considered as a pathogenic mutation [36]. Garmezy et al. also found pathogenic missense or frameshift mutations outside the exonuclease domain of *POLD1* [37].

### The relationship between the *POLE/POLD1* mutation and common tumors

#### The *POLE/POLD1* mutation and endometrial carcinoma

As early as 2013, TCGA classified *POLE*-mutant EC as a special molecular type and showed that *POLE*-mutant status is present in 10% of EC patients [38]. The somatic mutation rate of *POLE* is approximately 6.9%, and it usually shows MSS. Roberts et al. detected 30 *POLE* mutations in 535 cases of EC (5.6%) [39]. Church et al. found that 48 of 788 cases of EC (6.1%) had *POLE* mutations [40]. In FIG03 EC, the mutation rate of *POLE* is 15–22% [18, 26, 38]. In addition, common gene mutations in EC such as *PTEN*, *PIK3R1*, *PIK3CA*, *FBXW7*, and *KRAS* have a high mutation rate in this type of EC [38]. Castillo et al. found five hotspot mutations in a data analysis of ECs, which confirmed that mutations at different loci of *POLE* can have different functional effects [34]. The researchers also explored the complex relationship between *POLE* hotspot mutation and dMMR/MSI-H in EC. EC with both *POLE* hotspot mutation and MSI-H is rare, occurring only in 4.3% of cases. However, the associated TMB is high (median TMB is 339.0 mut/MB), which is consistent with that of cases with both *POLE* hotspot mutation and MSS. The median TMB of tumors with *POLE* non-hotspot mutation and MSI is 207.1 mut/MB. The median TMB of EC with *POLE* non-exonuclease domain mutation and MSI is 48.5 mut/MB [34]. Therefore, different *POLE* mutation sites can lead to different degrees of tumor mutation load. In another study of 173 cases of EC, 13 cases had *POLE* mutations, including 6 cases of P286R mutation and 2 cases of V411L mutation [25]. V411L is usually independent of the catalytic site, and its mutation may lead to tumorigenesis through secondary changes in the configuration of the DNA binding domain. P286R is a stronger missense mutation than V411L, which further indicates that mutations at different sites of *POLE* may lead to different somatic mutation profiles [40]. The *POLD1* mutation is very rare in EC. Briggs et al. showed that women with *POLD1* S478N mutation had a significantly increased risk of EC [17].

*POLE*-mutant EC is a unique clinical entity with a high immune response and good clinical prognosis. TCGA reported that patients with *POLE*-mutant EC had a younger age of onset and lower recurrence and mortality rates [38]. Billingsley et al. found that among 534 EC patients, 30 (5.6%) had *POLE* mutations and one recurred, with a recurrence rate of 3.4%, whereas the recurrence rate of *POLE*-wild-type patients was 17% [27]. Similarly, Church et al. showed that the recurrence and mortality rates of *POLE*-mutant EC patients were significantly lower than those of *POLE*-wild-type patients [40]. *POLE*-mutant ECs have a young age of onset (< 60 years old) and are mainly found in stage I EC patients. The mutation characteristics are different from those of MSI and are related to a good clinical prognosis [39]. EC with *POLE* hotspot mutation has a good prognosis and can achieve better progression free survival. It can be used as a prognostic molecular marker to guide the treatment of patients with grade 3 EC [41, 42].

#### *The POLE/POLD1* mutation and colorectal cancer

CRC is one of the most common malignant tumors of the digestive system. Zhu et al. confirmed that *POLE* mutations were associated with increased risk of gastrointestinal tumors, and *POLE* germline mutation might be an effective molecular marker for predicting survival and metastasis [43]. The incidence of somatic mutations in the exonuclease domain of *POLE* in CRC is approximately 3%, which is higher than that of germline mutations [44]. In a study of 6517 CRC patients, Glaire et al. found that *POLE* somatic mutations are more common in men, the right colon, and early stage patients, and are associated with a good prognosis [45]. Kane et al. found that the *POLE* P286R somatic mutation occurs in human CRC. The site of P286R is close to motif I, which is located in amino acids 271–285 and is necessary for the exonuclease function, as well as being next to the DNA binding site. Therefore, the P286R mutation affects the binding of the exonuclease domain to DNA and the proofreading function [46]. Palles et al. detected the L424V and *POLE* S478N germline mutations in multiple colorectal adenoma and CRC [47]. L424V is a conserved site in the exonuclease domain of the DNA polymerase B family. The germline mutation can reduce the fidelity of replication related polymerase proofreading and generate base substitutions, resulting in an increase in mutation rate and eventually leading to tumor occurrence [47]. Therefore, germline mutations can easily develop into multiple colorectal adenomas and CRC. The *POLD1* serine 478 is highly conserved in eukaryotes. The characteristics of colorectal tumors with *POLD1* S478N mutation are basically similar to those with the *POLE* L424V mutation [47]. Although the incidence of germline mutations in exonuclease domain of *POLE* in familial colorectal adenoma or CRC is only 0.1– 0.25%, inheritance of *POLE* germline mutations significantly increases the risk of CRC [48, 49]. Rohlin et al. found *POLE* Asn363Lys germline mutation in another hereditary tumor family. This family not only has inherited CRC, but also a high risk of EC, ovarian cancer, brain tumors, and other extraintestinal tumors. This site plays an important role in the binding of the exonuclease to substrates and is considered to be more oncogenic than the L424V mutation, thereby potentially leading to a variety of tumors [50].

There are few studies addressing the relation between *POLE* mutation and the prognosis of CRC patients. In 2016, Domingo et al. showed that mutant *POLE* CRC had a younger age of onset than wild-type *POLE* CRC, which was an independent prognostic factor relative to *BRAF* and *KRAS* mutations [45]. Other studies showed no significant difference in prognosis between *POLE*-mutant and *POLE*-wild-type CRC patients. However, the mortality of *POLE*-mutant patients who reach stage 3 or above and who are treated with adjuvant or palliative chemotherapy is significantly higher than that of *POLE*-wild-type CRC patients [51]. The effect of *POLE* mutation on the prognosis of CRC patients remains unclear, and further research is needed.

#### The *POLE/POLD1* mutation and non-small cell lung cancer

Song et al. analyzed 319 NSCLC patients and found that 2.8% of patients had *POLE* mutations [52]. Tobacco smoke, ultraviolet radiation, and alkylating agents are associated with high mutation load in NSCLC patients. Ultraviolet-related characteristics are almost only found in the squamous cell carcinoma subtype [20]. Min et al. reported that the V1446fs frameshift mutation of *POLE* was the site associated with the highest incidence of NSCLC (56.8%) [53]. In a study of the Chinese population, P286R and F699Vfs*11 were identified as the hotspot mutations of *POLE* in lung cancer, whereas the hotspot mutations recorded in the COSMIC database are P286R and V411L. P286R is the most common mutation in both the Chinese population and the COSMIC database, whereas F699Vfs*11 may be the most frequent mutation in the Chinese population. F699Vfs*11 is a frameshift mutation in the DNA polymerase type B ε subfamily catalytic domain that leads to the insertion of a termination codon, resulting in truncated, immature, or nonfunctional proteins. According to its location and type, F699Vfs*11 is generally considered a destructive mutation that may result in the destruction of a key polymerase domain and damage the function of the entire protein [54]. Due to the limited number of *POLD1* mutations, no hot spot changes of *POLD1* was found. The current analysis showed that the *POLE* mutation may be through the MMR, TGF-β, and RTK/RAS/RAF signaling pathways affecting tumor development, while *the POLD1* mutation may affect tumor development through the MMR signaling pathway [55].

The expression of TMB, PD-L1, and CD8 + tumor infiltrating lymphocyte is higher in *POLE*-mutant NSCLC patients than in *POLE*-wild-type patients. Therefore, POLE mutations may be candidate biomarkers for immunotherapy response in NSCLC patients [52]. However, there are important clinical differences between lung squamous cell carcinoma and lung adenocarcinoma. Liu et al. analyzed data of 513 patients with adenocarcinoma and 497 patients with squamous cell carcinoma from TCGA cohort, and the results suggested that *POLE* mutation is an effective biomarker of improved OS in patients with lung squamous cell carcinoma, whereas it has no effect on OS in patients with lung adenocarcinoma [56]. Further analysis of the lung adenocarcinoma population showed that patients with both PD-L1 overexpression and *POLE* mutation have significantly better OS [57]. Data from previous studies suggest that DNA repair status and TMB of lung adenocarcinoma could be used as independent biomarkers. Zhang et al. also confirmed that high expression of POLD1 was associated with poor prognosis of lung adenocarcinoma [58]. In a study of lung squamous cell carcinoma, Chae et al. confirmed that mutations in the DNA repair pathway are associated with high TMB [59]. However, these two subtypes are often grouped together in immunotherapy research, and few studies have explored their potential differences in the response to ICIs. The association between DNA repair pathway mutations and the treatment of different subtypes of NSCLC needs to be confirmed in a large number of basic studies.

POLE2, the second largest subunit of the Pol ε family, is involved in various cell functions, and *POLE2* mutations can affect cell repair, replication, and cell cycle regulation. Li et al. suggested that *POLE2* promotes NSCLC proliferation and growth. This study verified the oncogenic effect of *POLE2* on NSCLC using biological tests [57]. A study found that β-elemene inhibited the proliferation of lung cancer cells, and gene chip assays were used to detect β-elemene treated A549 cells. The results showed that intracellular *POLE2* was the most significantly downregulated gene, and deletion of *POLE2* inhibited proliferation and colony formation in A549 and NCI-H1299 cells, thus inducing apoptosis of tumor cells. Silencing of *POLE2* inhibited the growth, proliferation, and apoptosis of tumor cells in vitro [57].

#### The *POLE/POLD1* mutation and other solid tumors

Studies show that POLE2 is highly expressed in patients with esophageal squamous cell carcinoma (ESCC). In vivo and in vitro experiments show that POLE2 downregulation inhibits cancer cell proliferation and migration and promotes apoptosis. High expression of *POLE2* predicts tumor deterioration and poor prognosis in patients with ESCC, suggesting that *POLE2* is a potential therapeutic target for preventing or delaying the progression of ESCC [60]. In addition, gastric adenocarcinoma patients with *POLE/POLD1* mutations usually show adaptive immune resistance to the tumor microenvironment, loss of mismatch repair protein, increased PD-L1 expression, and higher TMB, suggesting that the *POLE/POLD1* mutation could be used as a biomarker to improve the clinical efficiency of precision medicine in gastric adenocarcinoma patients [61].

*POLE* S297F somatic mutation is detected in ovarian cancer. Unlike the common P286R and V411L mutations, S297F interacts with site 275 of the exonuclease catalytic region and affects the structure of the active site [62]. Detection of *POLE* hotspot mutations (P286R and V411L) in 251 samples of different subtypes of ovarian cancer in China showed that the *POLE* S297F mutation is more common in ovarian endometrioid carcinoma than other types of mutations, supporting that the *POLE* S297F mutation may actively participate in the pathogenesis of ovarian endometrioid carcinoma [62]. In 2018, Bhangoo et al. proposed that the detection of *POLE* mutation and TMB could improve the therapeutic potential of ICIs in patients with refractory uterine carcinosarcoma [63]. In addition, familial nonmuscular invasive bladder cancer caused by a G178R germline mutation of *POLD1* has also been reported, which has enriched the newly discovered genetic mutation pattern of this disease [64].

### The *POLE/POLD1* mutation, immunotherapy and prognosis

A high TMB is considered a molecular marker of the response to immunotherapy. Its mechanism is related to increased tumor immunogenicity and T cell activity caused by a high TMB. At present, there are few studies on the correlation between *POLD1* and the efficacy of ICI, and most of them focused on *POLE* mutations. Pathogenic mutations of *POLE* are closely related to a high TMB. In a correlation analysis of *POLE*-EDM and immune-related gene expression in TCGA [54, 65], Mo et al. found that *POLE*-EDM was positively correlated with significant CD8 + cytotoxic T lymphocyte (CTL) infiltration, and cytotoxic T cell effect markers were significantly upregulated. Further studies showed that CD8 + CTL, CD45RO + memory immune cells (MIC), and CD8 + CD45RO + MIC were significantly more abundant in *POLE*-EDMs than in *POLE*-wild-type and *POLE* non-exonuclease type. Further combined with MSI status, CD8 + CTL, CD45RO + MIC, and CD8 + CD45RO + MIC in MSI-H tumors were lower than those in *POLE*-EDM tumors, but higher than those in MSS. This suggest that regardless of MSI status, *POLE*-EDM tumors have a stronger T lymphocyte infiltration ability and a stronger antitumor activity [28].

To determine the effect of *POLE/POLD1* mutation on immunotherapy efficacy, Wang et al. collected and analyzed gene detection data of 47,721 patients with different types of cancer. Multivariate Cox regression analysis after adjustment for MSI status and tumor type showed that *POLE/POLD1* is an independent risk factor for immunotherapy benefit [21]. Other studies showed that the expression of immune checkpoint related proteins such as PD-1 and cytotoxic T lymphocyte associated antigen-4 (CTLA-4) is increased in *POLE*-mutated EC, and the number of tumor-infiltrating T cells is high. One possible underlying mechanism is that *POLE* mutations lead to tumor hypermutation and increase the expression of tumor neoantigens [66, 67]. In 2015, Howitt et al. showed that the tumor neoantigen produced by EC with *POLE* mutation is 15-fold higher than that of MSI and more than 100-fold higher than that of MSS [68].

*POLE-*EDM has a positive effect on prognosis. Studies show that only a small proportion of somatic mutations in tumor cells are driver mutations, resulting in the inability of cancer cells to complete normal differentiation, thus becoming malignant proliferating cells characterized by uncontrolled growth and division. In addition, most of the mutations are passenger mutations. In the past, passenger mutations were thought to be unrelated to the occurrence and progression of tumors. However, in recent years, studies have shown that accumulated passenger mutations can destroy tumor cells, slow down their growth, and even promote cell death through different mechanisms, such as the production of toxic proteins, induction of immune responses, or loss of tumor cell function. Loss of the proofreading function of *POLE*-mutant tumors leads to a large number of wrong bases produced during replication. Passenger mutations that accumulate in cells may lead to the slow growth and even death of tumor cells with *POLE* mutation, which may underlie the favorable prognosis of *POLE*-mutant tumors. Some studies have explored the prognosis of patients with *POLE* mutation treated with ICIs. Rizvi et al. reported that four NSCLC patients with *POLE* mutation achieved a progression free survival of 8–14 months after treatment with pembrolizumab, suggesting that the efficacy of anti-PD-1 therapy is higher in NSCLC with *POLE* mutation [69]. Min et al. also showed that the level of infiltrating T cells in *POLE*-mutated NSCLC is increased, resulting in a favorable prognosis [53]. The KEYNOTE-028 study reported that a *POLE*-mutated patient with advanced EC achieved partial remission after 8 weeks of treatment with pembrolizumab and achieved continuous remission for more than 14 months [70]. Other studies have found that patients with *POLE* mutations outside the exonuclease domain could still have a lasting response to ICI, through the analysis of multiple large genome data sets. An analysis of 1,278 patients with advanced cancer with low/moderate TMB treated with ICIs showed that the occurrence of *POLE* missense mutations outside the exonuclease domain was associated with better overall survival [36]. Therefore, although the *POLE* mutations outside the exonuclease domain do not lead to high mutations, they may enhance immunogenicity through a mechanism independent of new antigens.

## Conclusions

*POLE/POLD1* is involved in the development of multiple tumors. Patients with *POLE/POLD1*-mutated tumors are characterized by a young age and good prognosis, suggesting the potential of *POLE/POLD1* mutation as a new indicator of prognosis. *POLE/POLD1* mutation is closely related to a high mutation load, increased neoantigens, and increased intracellular immune cell infiltration in tumors. However, whether it is a potential biomarker to predict the efficacy of immunotherapy, needs more clinical data. Relevant clinical studies are ongoing (NCT05103969, NCT03810339). But, the incidences of *POLE/POLD1* mutations are low and their locations differ, which may be a dominant factor in its clinical limitations. In addition, studies on *POLE/POLD1* mutations have not been extended to all tumor types, and there are relatively few studies on the morphological and immunohistochemical expression rates of *POLE/POLD1*-mutated tumors. Furthermore, the differences in *POLE/POLD1* mutation frequency among different regions and races need to be further verified. Further multi-center prospective clinical studies are needed to identify new diagnostic and treatment methods for *POLE/POLD1*-mutant tumors.

## Data Availability

The references supporting the conclusions of this article is included within the article.

## Abbreviations

ICI: Immune checkpoint inhibitors
PD-1: Programmed death receptor 1
NSCLC: Non-small cell lung cancer
dMMR: Deficient mismatch repair
MSI-H: High microsatellite instability
ORR: Objective response rate
TMB-H: High tumor mutation burden
OS: Overall survival
Pol ε: Polymerase ε
Pol δ: Polymerase δ
*POLE*/*POLD1*-EDM: Exonuclease domain mutation of *POLE*/*POLD1*
EC: Endometrial cancer
CRC: Colorectal cancer
MSS: Microsatellite stability
TCGA: The Cancer Genome Atlas
ESCC: Esophageal squamous cell carcinoma
CIL: Cytotoxic T lymphocyte
MIC: Memory immune cell
CTLA-4: Cytotoxic T lymphocyte associated antigen-4

## References

[1] Sung H, Ferlay J, Siegel RL (2021). Global Cancer Statistics 2020: GLOBOCAN Estimates of Incidence and Mortality Worldwide for 36 Cancers in 185 Countries. CA Cancer J Clin.

[2] Gettinger S, Horn L, Jackman D (2018). Five-Year Follow-Up of Nivolumab in Previously Treated Advanced Non-Small-Cell Lung Cancer: Results From the CA209-003 Study. J Clin Oncol..

[3] Topalian SL, Hodi FS, Brahmer JR (2012). Safety, activity, and immune correlates of anti-PD-1 antibody in cancer. N Engl J Med.

[4] Marcus L, Lemery SJ, Keegan P (2019). FDA Approval Summary: Pembrolizumab for the Treatment of Microsatellite Instability-High Solid Tumors. Clin Cancer Res..

[5] Le DT, Durham JN, Smith KN (2017). Mismatch repair deficiency predicts response of solid tumors to PD-1 blockade. Science.

[6] Baretti M, Le DT (2018). DNA mismatch repair in cancer. Pharmacol Ther.

[7] Chung HC, Ros W, Delord JP (2019). Efficacy and Safety of Pembrolizumab in Previously Treated Advanced Cervical Cancer: Results From the Phase II KEYNOTE-158 Study. J Clin Oncol..

[8] Strosberg J, Mizuno N, Doi T (2020). Efficacy and Safety of Pembrolizumab in Previously Treated Advanced Neuroendocrine Tumors: Results From the Phase II KEYNOTE-158 Study. Clin Cancer Res..

[9] Gibney GT, Weiner LM, Atkins MB (2016). Predictive biomarkers for checkpoint inhibitor-based immunotherapy. Lancet Oncol.

[10] Wu HX, Chen YX, Wang ZX (2019). Alteration in TET1 as potential biomarker for immune checkpoint blockade in multiple cancers. J Immunother Cancer..

[11] Havel JJ, Chowell D, Chan TA (2019). The evolving landscape of biomarkers for checkpoint inhibitor immunotherapy. Nat Rev Cancer.

[12] Preston BD, Albertson TM, Herr AJ (2010). DNA replication fidelity and cancer. Semin Cancer Biol.

[13] Korona DA, Lecompte KG, Pursell ZF (2011). The high fidelity and unique error signature of human DNA polymerase epsilon. Nucleic Acids Res.

[14] Loeb LA, Monnat RJ (2008). DNA polymerases and human disease. Nat Rev Genet..

[15] Lehmann AR (2011). DNA polymerases and repair synthesis in NER in human cells. DNA Repair (Amst).

[16] Lydeard JR, Jain S, Yamaguchi M (2007). Break-induced replication and telomerase-independent telomere maintenance require Pol32. Nature..

[17] Briggs S, Tomlinson I (2013). Germline and somatic polymerase ε and δ mutations define a new class of hypermutated colorectal and endometrial cancers. J Pathol.

[18] Rayner E, van Gool IC, Palles C (2016). A panoply of errors: polymerase proofreading domain mutations in cancer. Nat Rev Cancer.

[19] Yoshida R, Miyashita K, Inoue M (2011). Concurrent genetic alterations in DNA polymerase proofreading and mismatch repair in human colorectal cancer. Eur J Hum Genet.

[20] Albertson TM, Ogawa M, Bugni JM (2009). DNA polymerase epsilon and delta proofreading suppress discrete mutator and cancer phenotypes in mice. Proc Natl Acad Sci U S A.

[21] Li HD, Cuevas I, Zhang M (2018). Polymerase-mediated ultramutagenesis in mice produces diverse cancers with high mutational load. J Clin Invest..

[22] Wang F, Zhao Q, Wang YN (2019). Evaluation of POLE and POLD1 Mutations as Biomarkers for Immunotherapy Outcomes Across Multiple Cancer Types. JAMA Oncol..

[23] Hwang HS, Kim D, Choi J (2021). Distinct mutational profile and immune microenvironment in microsatellite-unstable and POLE-mutated tumors. J Immunother Cancer.

[24] Chung J, Maruvka YE, Sudhaman S (2021). DNA Polymerase and Mismatch Repair Exert Distinct Microsatellite Instability Signatures in Normal and Malignant Human Cells. Cancer Discov.

[25] Carethers JM, Jung BH (2015). Genetics and Genetic Biomarkers in Sporadic Colorectal Cancer. Gastroenterology.

[26] Church DN, Briggs SE, Palles C (2013). DNA polymerase ε and δ exonuclease domain mutations in endometrial cancer. Hum Mol Genet..

[27] Meng B, Hoang LN, McIntyre JB (2014). POLE exonuclease domain mutation predicts long progression-free survival in grade 3 endometrioid carcinoma of the endometrium. Gynecol Oncol.

[28] Billingsley CC, Cohn DE, Mutch DG (2015). Polymerase ɛ (POLE) mutations in endometrial cancer: clinical outcomes and implications for Lynch syndrome testing. Cancer..

[29] Mo S, Ma X, Li Y (2020). Somatic POLE exonuclease domain mutations elicit enhanced intratumoral immune responses in stage II colorectal cancer. J Immunother Cancer.

[30] Vasaikar S, Huang C, Wang X (2019). Proteogenomic Analysis of Human Colon Cancer Reveals New Therapeutic Opportunities. Cell..

[31] Haradhvala NJ, Kim J, Maruvka YE (2018). Distinct mutational signatures characterize concurrent loss of polymerase proofreading and mismatch repair. Nat Commun..

[32] Temko D, Van Gool IC, Rayner E (2018). Somatic POLE exonuclease domain mutations are early events in sporadic endometrial and colorectal carcinogenesis, determining driver mutational landscape, clonal neoantigen burden and immune response. J Pathol.

[33] Zhu B, Liu Y, Li J (2020). Exceptional Response of Cryoablation Followed by Pembrolizumab in a Patient with Metastatic Cervical Carcinosarcoma with High Tumor Mutational Burden: A Case Report. Oncologist.

[34] He J, Ouyang W, Zhao W (2021). Distinctive genomic characteristics in POLE/POLD1-mutant cancers can potentially predict beneficial clinical outcomes in patients who receive immune checkpoint inhibitor. Ann Transl Med.

[35] León-Castillo A, Britton H, McConechy MK (2020). Interpretation of somatic POLE mutations in endometrial carcinoma. J Pathol.

[36] Dong S, Zakaria H, Hsiehchen D (2022). Non-Exonuclease Domain POLE Mutations Associated with Immunotherapy Benefit. Oncologist.

[37] Garmezy B, Gheeya J, Lin HY (2022). Clinical and Molecular Characterization of POLE Mutations as Predictive Biomarkers of Response to Immune Checkpoint Inhibitors in Advanced Cancers. JCO Precis Oncol.

[38] Kandoth C, Schultz N, Cherniack AD, Cancer Genome Atlas Research Network (2013). Integrated genomic characterization of endometrial carcinoma. Nature.

[39] Roberts SA, Gordenin DA (2015). Hypermutation in human cancer genomes: footprints and mechanisms. Nat Rev Cancer..

[40] Church DN, Stelloo E, Nout RA (2014). Prognostic significance of POLE proofreading mutations in endometrial cancer. J Natl Cancer Inst..

[41] Wang Y, Yu M, Yang JX (2019). Genomic Comparison of Endometrioid Endometrial Carcinoma and Its Precancerous Lesions in Chinese Patients by High-Depth Next Generation Sequencing. Front Oncol.

[42] Akhtar M, Al Hyassat S, Elaiwy O (2019). Classification of Endometrial Carcinoma: New Perspectives Beyond Morphology. Adv Anat Pathol.

[43] Castellsagué E, Li R, Aligue R (2019). Novel POLE pathogenic germline variant in a family with multiple primary tumors results in distinct mutational signatures. Hum Mutat.

[44] Cancer Genome Atlas Network (2012). Comprehensive molecular characterization of human colon and rectal cancer. Nature.

[45] Domingo E, Freeman-Mills L, Rayner E (2016). Somatic POLE proofreading domain mutation, immune response, and prognosis in colorectal cancer: a retrospective, pooled biomarker study. Lancet Gastroenterol Hepatol.

[46] Kane DP, Shcherbakova PV (2014). A common cancer-associated DNA polymerase ε mutation causes an exceptionally strong mutator phenotype, indicating fidelity defects distinct from loss of proofreading. Cancer Res..

[47] Palles C, Cazier JB, Howarth KM (2013). Germline mutations affecting the proofreading domains of POLE and POLD1 predispose to colorectal adenomas and carcinomas. Nat Genet..

[48] Valle L, Hernández-Illán E, Bellido F (2014). New insights into POLE and POLD1 germline mutations in familial colorectal cancer and polyposis. Hum Mol Genet..

[49] Elsayed FA, Kets CM, Ruano D (2015). Germline variants in POLE are associated with early onset mismatch repair deficient colorectal cancer. Eur J Hum Genet.

[50] Rohlin A, Zagoras T, Nilsson S (2014). A mutation in POLE predisposing to a multi-tumour phenotype. Int J Oncol.

[51] Stenzinger A, Pfarr N, Endris V (2014). Mutations in POLE and survival of colorectal cancer patients–link to disease stage and treatment. Cancer Med.

[52] Song Z, Cheng G, Xu C (2018). Clinicopathological characteristics of POLE mutation in patients with non-small-cell lung cancer. Lung Cancer.

[53] Min KW, Kim WS, Kim DH (2020). High polymerase ε expression associated with increased CD8 + T cells improves survival in patients with non-small cell lung cancer. PLoS ONE.

[54] Yao J, Gong Y, Zhao W (2019). Comprehensive analysis of POLE and POLD1 Gene Variations identifies cancer patients potentially benefit from immunotherapy in Chinese population. Sci Rep..

[55] Yao J, Gong Y, Zhao W (2019). Comprehensive analysis of POLE and POLD1 Gene Variations identifies cancer patients potentially benefit from immunotherapy in Chinese population. Sci Rep..

[56] Liu L, Ruiz J, O’Neill SS (2018). Favorable outcome of patients with lung adenocarcinoma harboring POLE mutations and expressing high PD-L1. Mol Cancer..

[57] Li J, Wang J, Yu J (2018). Knockdown of POLE2 expression suppresses lung adenocarcinoma cell malignant phenotypes in vitro. Oncol Rep.

[58] Zhang L, Chen J, Yang H (2021). Multiple microarray analyses identify key genes associated with the development of Non-Small Cell Lung Cancer from Chronic Obstructive Pulmonary Disease. J Cancer..

[59] Chae YK, Anker JF, Oh MS (2019). Mutations in DNA repair genes are associated with increased neoantigen burden and a distinct immunophenotype in lung squamous cell carcinoma. Sci Rep..

[60] Zhu Y, Chen G, Song Y (2020). POLE2 knockdown reduce tumorigenesis in esophageal squamous cells. Cancer Cell Int.

[61] Zhu M, Cui H, Zhang L (2022). Assessment of POLE and POLD1 mutations as prognosis and immunotherapy biomarkers for stomach adenocarcinoma. Transl Cancer Res.

[62] Zou Y, Liu FY, Liu H (2014). Frequent POLE1 p.S297F mutation in Chinese patients with ovarian endometrioid carcinoma. Mutat Res.

[63] Bhangoo MS, Boasberg P, Mehta P (2018). Tumor Mutational Burden Guides Therapy in a Treatment Refractory POLE-Mutant Uterine Carcinosarcoma. Oncologist..

[64] Wang Y, Ju L, Guo Z (2021). Pedigree analysis of a POLD1 germline mutation in urothelial carcinoma shows a close association between different mutation burdens and overall survival. Cell Mol Immunol.

[65] Fumet JD, Truntzer C, Yarchoan M (2020). Tumour mutational burden as a biomarker for immunotherapy: Current data and emerging concepts. Eur J Cancer.

[66] van Gool IC, Bosse T, Church DN (2015). POLE proofreading mutation, immune response and prognosis in endometrial cancer. Oncoimmunology..

[67] van Gool IC, Eggink FA, Freeman-Mills L (2015). POLE Proofreading Mutations Elicit an Antitumor Immune Response in Endometrial Cancer. Clin Cancer Res..

[68] Howitt BE, Shukla SA, Sholl LM (2015). Association of Polymerase e-Mutated and Microsatellite-Instable Endometrial Cancers With Neoantigen Load, Number of Tumor-Infiltrating Lymphocytes, and Expression of PD-1 and PD-L1. JAMA Oncol.

[69] Rizvi NA, Hellmann MD, Snyder A (2015). Cancer immunology. Mutational landscape determines sensitivity to PD-1 blockade in non-small cell lung cancer. Science..

[70] Ott PA, Bang YJ, Berton-Rigaud D (2017). Safety and Antitumor Activity of Pembrolizumab in Advanced Programmed Death Ligand 1-Positive Endometrial Cancer: Results From the KEYNOTE-028 Study. J Clin Oncol..

